# Pollen Lipidomics: Lipid Profiling Exposes a Notable Diversity in 22 Allergenic Pollen and Potential Biomarkers of the Allergic Immune Response

**DOI:** 10.1371/journal.pone.0057566

**Published:** 2013-02-28

**Authors:** Mohamed Elfatih H. Bashir, Jan Hsi Lui, Ravishankar Palnivelu, Robert M. Naclerio, Daphne Preuss

**Affiliations:** 1 Section of Otolaryngology-Head and Neck Surgery, Department of Surgery, Division of the Biological Sciences, The University of Chicago, Chicago, Illinois, United States of America; 2 Department of Molecular Genetics and Cell Biology, Division of the Biological Sciences, The University of Chicago, Chicago, Illinois, United States of America; 3 The School of Plant Sciences, University of Arizona, Tucson, Arizona, United States of America; 4 Chromatin, Inc., Chicago, Illinois, United States of America; BASF Cropdesign, Belgium

## Abstract

**Background/Aim:**

Pollen grains are the male gametophytes that deliver sperm cells to female gametophytes during sexual reproduction of higher plants. Pollen is a major source of aeroallergens and environmental antigens. The pollen coat harbors a plethora of lipids that are required for pollen hydration, germination, and penetration of the stigma by pollen tubes. In addition to proteins, pollen displays a wide array of lipids that interact with the human immune system. Prior searches for pollen allergens have focused on the identification of intracellular allergenic proteins, but have largely overlooked much of the extracellular pollen matrix, a region where the majority of lipid molecules reside. Lipid antigens have attracted attention for their potent immunoregulatory effects. By being in close proximity to allergenic proteins on the pollen surface when they interact with host cells, lipids could modify the antigenic properties of proteins.

**Methodology/Principal Findings:**

We performed a comparative pollen lipid profiling of 22 commonly allergenic plant species by the use of gas chromatography-mass spectroscopy, followed by detailed data mining and statistical analysis. Three experiments compared pollen lipid profiles. We built a database library of the pollen lipids by matching acquired pollen-lipid mass spectra and retention times with the NIST/EPA/NIH mass-spectral library. We detected, identified, and relatively quantified more than 106 lipid molecular species including fatty acids, n-alkanes, fatty alcohols, and sterols. Pollen-derived lipids stimulation up-regulate cytokines expression of dendritic and natural killer T cells co-culture.

**Conclusions/Significance:**

Here we report on a lipidomic analysis of pollen lipids that can serve as a database for identifying potential lipid antigens and/or novel candidate molecules involved in allergy. The database provides a resource that facilitates studies on the role of lipids in the immunopathogenesis of allergy. Pollen lipids vary greatly among allergenic species and contain many molecules that have stimulatory or regulatory effects on immune responses.

## Introduction

Asthma and allergic diseases are among the major causes of illness and disability in the United States, with young children being especially vulnerable because of their immature immune systems. Pollen allergy has a remarkable clinical impact, affecting more than 25% of the population. Allergic rhinitis (AR) is responsible for a substantial healthcare cost burden, estimated to be more than $15 billion annually [1].

Pollen grains are the male gametophytes that deliver sperm cells to female gametophytes during sexual reproduction of higher plants. The surface of a dehydrated pollen grain consists of three layers: the inner wall (intine), the outer wall (exine), and the extracellular matrix (the pollen coat or surface) [10]. The pollen coat contains lipids that are required for pollen hydration, germination, and penetration of the stigma by pollen tubes [2]–[6]. Prior searches for pollen allergens have focused on the identification of intracellular allergenic proteins inducing IgE responses [7], [8], but have largely overlooked much of the extracellular pollen matrix, a region where lipidic molecules that are potentially immunogenic reside. The essential role played by lipids in pollen-stigma recognition and interaction during the initial steps of fertilization is well understood [2], [9], [10]. Very-long-chain lipids contribute to the hydrophobic cuticle on the surface of all higher plants and are an indispensable component of the extracellular pollen coat in the Brassicaceae [4], [9]. In *Arabidopsis,* the loss of pollen-coat lipids can disrupt cell signaling with the stigma during fertilization, inhibiting pollen hydration and causing sterility [4], [9], [11].

Lipid molecular species derived from plants are known to cause inflammation and allergic contact dermatitis. These molecules include urushiol, a phenolic lipid from *Toxicodendron radicans* (poison ivy) [12], [13], and falcarinol, a 17-carbon alkene from *Hedera helix* (English ivy) [14]. What makes pollen such a potent allergen? By being in close proximity to allergenic proteins on the pollen surface when they interact with host cells, lipids could modify the antigenic properties of proteins. This proximity also raises the possibility of a “two-hit” signal composed of pollen proteins (allergens) and pollen lipophilic components (adjuvants) to initiate an allergic response and provide synergy. Given that lipids are critical members of cellular signal transduction pathways, it is conceivable that pollens form a rich source of immune-stimulatory molecules that may play a potential role in the immune-regulation and control allergic response.

Natural killer T (NKT) cells are specialized T cells of the immune system that express markers of the NK cell lineage, such as NK1.1. In the mouse, these cells are sometimes referred to as invariant NKT (iNKT) cells, because they express a semi-invariant T cell receptor (TCR) consisting of a single Vα-to-Jα rearrangement (Vα14-Jα18), paired with a restricted set of Vβ chains (Vβ8.2, Vβ7, and Vβ2 are the most common). NKT cells are important in a variety of immune responses through the rapid and substantial secretion of T-helper type 1 (T_H_1) and T_H_2 cytokines. Unlike other T cells, NKT cell are restricted to a non-major histocompatibility complex (MHC) molecule, CD1d, which binds lipids instead of proteins [15]. NKT cell produce very large amounts of cytokines within 1–2 h of primary stimulation and without the need for clonal expansion. They can drive immune responses in either the pro- or anti-inflammatory direction, thus promoting or suppressing acquired immunity.

Traditionally, lipids are regarded as inactive molecules embedded in membranes that may carry bioactive molecules. However, it is becoming evident that lipids themselves are critical molecules involved in immune responses. Pollen lipids from cypress are recognized as antigens by human T cells through a CD1-dependent pathway [16] and liberate bioactive lipid mediators [17], [18], [19]. CD1-restricted γ-δ T cells specific for pollen phospholipids have been associated with the mucosal regulatory response against tree pollens [16], [20]. The lipid-activated transcription factor peroxisome proliferator-activated receptor γ (PPARγ) promotes the development of a specific dendritic cell (DC) subtype [21]. Agonists of PPARγ up-regulate CD1d molecules linked to NKT cell activation, and they affect the function of lymphoid cells *in vitro* [22], [23], [24], [25]. Hydrocarbons play a critical role in antigen-specific immunoadjuvanticity by affecting the onset, magnitude, and duration of immune responses [26] and allergic respiratory diseases [27]. Whereas recent studies indicate a role of lipids in the immune response, there remains insufficient rigorous evidence to address the stimulatory role of pollen lipids in allergic response*s*.

In contrast to the role of pollen lipids in pollen-stigma interaction, the role of pollen-lipid molecular mechanism(s) of early events of T_H_1 and T_H_2 polarization and allergic sensitization after inhalation of pollen are not well understood. The mechanism(s) governing iNKT-cell activation by CD1d and its ligands and their contribution to the T_H_2 polarizing program during pollen allergy are largely unknown.

Recently, lipidomics or lipid profiling, an extension of metabolomics that focuses on lipids, has received increasing attention as a research tool in several disciplines, including lipid biochemistry, pathology, and potential biomarker discovery. Despite accumulating evidence that underscores the importance of lipids in immune regulation, the number, identity, and abundance of pollen lipid molecular species have not been elucidated.

We undertook the current study to identify features of the lipid profiles of the pollen of allergenic plants by using a gas chromatography-mass spectroscopy (GC-MS)-based lipidomic approach combined with traditional immunologic methods. An immunologic assessment of the lipid-regulating effects of pollen-derived lipids was conducted by use of a DC/NKT co-culture *in vitro* assay for classification of the stimulatory activities of lipids. The results from the lipidomic approach were not only compared for pollen lipids from 22 highly allergenic species (n = 66) obtained with GC-MS, but they were also analyzed for identification of potential novel biomarkers and/or lipid antigens from these species in an effort further to explore the mechanisms of action of pollen lipids in allergic diseases. This lipidomic analysis is an attempt to remedy the lack of available information on these important allergenic species. The importance of this study is based on the identification of the finger-printing of allergenic pollen lipids and the possibility of developing assays for study of their role in allergy.

We also examined the mechanism of activation of CD1d-restricted iNKT cells in response to pollen lipid products *in vitro,* highlighting the pro-allergic and pro-inflammatory cytokines secreted by DCs co-cultured with CD1d-restricted iNKT cells after exposure to pollen-lipid products. This forms a necessary part of an ongoing series of investigations to establish whether any immunological roles, specifically those influencing the allergic response, can be attributed to these lipid compounds.

The data set generated here provides a thorough cataloguing of the number, identity, and relative levels of pollen-associated lipids in 22 pollen species, including novel lipid-antigen candidate molecules that are potentially involved in allergy. To the best of our knowledge, this work represents the first attempt to identify and survey pollen “lipidomes” and the lipid signature of several allergenic pollen species. The results provide baseline data for further studies on the possible role of pollen lipids in immuno-stimulation.

## Materials and Methods

### Ethics Approval

All animal care and experimental procedures were performed in accordance with NIH and USDA guidelines, and approved by the University of Chicago Institutional Animal Care and Use Committee.

### Pollen Sources

One of the most prominent features of pollen coat is the presence of a unique, lipid-rich layer covering the pollen surfaces. During the first stages of these investigations, collections of fresh pollen grains from plants grown under field conditions were challenging. We were unable to collect representative samples of the area pollen. When large number of allergenic pollen species grown at different climates is required for our analysis, it was impossible to control for contamination, quality of the source material and lot-to-lot consistency under field growing conditions. We arranged for our laboratory to receive fresh (not dried), raw, non-defatted batches of pollens that were stored under controlled and monitored condition at <0°C from Greer Laboratories, Lenoir, NC, USA. Pollen source (Genus/species) variability within the Greer laboratories is controlled by using reproducible procedures, single large lots of pollen source material. The pollens were collected from fields, processed and microscopically inspected to produce source material lots with high purity and confirmed identity.

Pollen from 6 grass species (Bermuda grass, *Cynodon dactylon*; Kentucky Blue/June grass, *Poa pratensis*; Johnson grass, *Sorghum halepense*; orchard grass, *Dactylis glomerata*; perennial rye, *Lolium perenne;* Timothy grass, *Phleum pretense);* 2 weed species (common mugwort, *Artemisia vulgaris;* short ragweed, *Ambrosia artemisiifolia*); and 14 tree species (European alder, *Alnus glutinosa;* white ash, *Fraxinus americana;* white birch, *Betula populifolia;* box elder, *Acer negundo;* mountain cedar, *Juniperus ashei* [sabinoides]; eastern cottonwood, *Populus deltoids*; American elm, *Ulmus americana*; red mulberry, *Morusrubra*; red oak, *Quercus rubra;* white oak, *Quercus alba*; olive, *Olea europaea;* pecan, *Carya illinoensis*; Western sycamore, *Platanus racemosa;* and black walnut, *Juglans nigra)* have been studied in this work.

All pollens were processed immediately after received from Greer laboratories (within hours) for GC-MS to ensure their integrity. The rest was stored at −20°C. We have conducted three separate sampling from each pollen species (n = 3×22) for lipid extraction experiments. We have statistically treated every sample as an independent variable. By extracting the pollen lipid from independent samples prior to solvent extraction and combining the average results, we have reproducibly gotten lipid extracts with consistent lipid molecular species.

### Reagents

The organic solvents used were of the highest purity available and were purchased from Sigma (St. Louis, MO, USA). All fatty acids (purity: minimum 99.6%), n-alkanes (purity: minimum 99.5%) and lipids were purchased from Sigma (St. Louis, MO, USA). The lipids are stored as recommended by the supplier. Stock solutions of all lipid compounds were prepared at concentration of 100 mM solution in dimethyl sulfoxide (DMSO):0.1% Tween-20 in PBS (1∶10, v/v). Working solutions of all lipophilic compounds and hydrocarbons were made up with 0.1% in PBS at varied concentrations in the range of 1–10 ug/ml, vortexed, homogenized and sonicated.

### Assessment of the Effects of Organic Solvents on Lipid Extractability and Pollen Hydration before Extraction of Pollen Lipophilic Components

In preliminary studies, our objective was to search out the effective organic solvent to extract pollen lipid from the pollen coat. The purpose of the studies was to compare organic solvents for recovery of different pollen lipids. The potential of 24 organic solvents to extract pollen coat material without affecting the hydration of the pollen was evaluated as described previously [28] with modification. Pollen from Bermuda grass, Ragweed, western Sycamore and American Elm were put separately into tubes and extracted with protic and aprotic solvents that ranged from non-polar to polar including: n-hexane, cyclohexane, benzene, chloroform, N, N-dimethylformamide, dimethyl sulfoxide, toluene, diethyl ether, petroleum ether, ethanol, methanol, acetone, and acetonitrile to obtain soluble lipids with varying polarity indices. Extraction of organic solvent soluble components in the pollen coat was performed very rapidly and consistently while trying to retain the integrity of the pollen coat and the cytoplasmic contents of the pollen. Each treatment tube was vortexed for 5 min after addition of the solvent. Aliquots of 100 µL from each solvent and pollen were examined by use of an inverted microscope. Bright-field images encompassing at least 100 pollen grains were photographed at 100× magnification for each treatment reaction after 30 min, 21 h and 7 days. Pollen was classified based upon specific morphologic characteristics. Non-hydrated pollen grains (NHPs) were classified by their oblong morphology, which resembles a deflated football, as opposed to round hydrated pollen. Comparisons of total lipids yield extracted from pollen with each solvent were made. The isolated hydrophobic proteins from BGP were reconstituted in PBS, and protein concentrations were determined by the use of a BCA kit (Pierce, Rockford, IL, USA) according to the manufacturer’s protocol. After 30 min, 21 h and 7 days of treatment, pollen grains were categorized into three classifications. The first class consisted of hydrated pollen. The second class consisted of grains that were partially hydrated. The third class was NHPs. Pollen hydration, lipid contents level, the classes of the lipid and protein concentrations assisted in identifying the solvents that extract enough pollen coat lipid and/or hydrophobic proteins without jeopardizing the integrity of the pollen surface or result in pollen hydration. Our strategy was to get as pure pollen lipid extract as possible and exclude solvents that also extract hydrophobic proteins. Exclusion of proteins from total lipid extract before GC/MS analysis is important to provide clean detection and interference free analysis. On the basis of these factors, we found that the type of organic solvent affect the total lipid extracted. Chloroform yielded greater total lipid content (lipid extractability) and the least hydration rates than the rest of organic solvents. Cyclohexane was found to be by far the best solvent for its high protein extractability and low pollen hydration rates (data not shown). We concluded that chloroform is the optimum extraction solvent which perfectly matched our needs and can be directly used for GC-MS-based lipidomic analysis. Extraction of pollen lipids with chloroform before GC/MS analysis provides clean detection and interference free analysis.

### Micro-scale Extraction of Pollen Lipids

A modified extraction technique for isolating pollen lipophilic components, without disrupting the pollen cell-wall integrity, was adopted from previously described reports [29], [30]. In brief, 250 milligrams of pollen were mixed with 1000 µl chloroform containing 100 µg of the internal standard, nonadecane (Sigma, St. Louis, MO), and vortexed for 5 minutes. Addition of the internal standard to the samples prior to extraction enables calculation of the derivatization efficiency. The mixture was centrifuged for 5 min at 2000 rpm at room temperature. The supernatant was carefully decanted to minimize contamination by particulate matter. The remaining total solid material was then extracted twice with fresh chloroform (1000 µL). The chloroform extracts were pooled, filtered through Whatman No. 54 filter paper, concentrated under nitrogen, and then transferred to 2-ml screw-cap vials. The extracts were dried under a gentle nitrogen stream at 25°C. The dry residue was resuspended in 100 µL of chloroform and vortexed vigorously (stock sample). Three independent samples were extracted from each species.

### Preparation of BSTFA Derivatives

Twenty µL of stock sample solution was transferred to 2-ml amber auto sampler vials, and the solution was dried with a stream of nitrogen. The residue was then derivatized by reconstituting with 80 µL of N, O-bis(trimethylsilyl)-trifluoroacetamide (BSTFA) and trimethylchlorosilane (99∶1, v/v, Supelco, Bellefonte, PA), in a total volume of 100 µL. The vials were capped and heated at 80°C for 20 minutes. The trimethylsilyl ether derivatives were dissolved in 50 µL of hexane and transferred to an autoinjector vial. One µL of this solution was injected in the GC-MS with an automatic liquid sampler.

### Gas Chromatography-Mass Spectrometry (GC-MS) Analysis

The chloroform-extracted lipids were derivatized and subjected to GC-MS qualitative and relative quantitative analysis at the Mass Spectrometry Facility of Michigan State University (East Lansing, MI). Experiments were carried out with an Agilent 6890N HP gas chromatograph (Agilent Technologies, Palo Alto, CA, USA) equipped with an MSD mass spectrometer. The column used was a DB5MS fused silica capillary column (60 m×0.25 mm ID and 0.25 µm film thickness; Supelco, Bellefonte, PA).

The integrated system was operated under an HP ChemStation (Agilent, Palo Alto, CA). The oven temperature was set at 50°C for 1 min, then increased to 300°C by 20°C/min, and held at 300°C for 5 min. The temperatures of the injection port and of the detector were set at 250°C and 280°C, respectively. The electron ionization voltage was 70 eV. Data were obtained in a full-scan mode with a scan range from 50 to 1200**m/z (mass-to-charge ratio).

### Identification, Relative Quantification of Lipid Compounds of GC-MS Data and Statistical Analysis

Data manipulation was performed with use of spreadsheets. Chromatogram peaks identified from the eluted lipid molecular species were based on unequivocal matches by comparison of the retention time and mass spectrum of each peak with the NIST/EPA/NIH Mass Spectral Library (National Institute of Standards and Technology, Gaithersburg, MD) and in-house databases. The NIST/EPA/NIH library contains more than 165,000 standard reference spectra of high quality. Mass-spectral deconvolution and automated calculation of retention time were performed with use of the automated mass spectral deconvolution and identification system (AMDIS) software version 2.6, (NIST, Gaithersburg, MD). Using GC-MS and AMDIS software, we constructed a comprehensive library of pollen lipid molecular species and generated a list of candidate lipid biomarkers either unique to one species or common across all of the pollen examined.

High-scoring matches were designated by the Union of Pure and Applied Chemistry (IUPAC) system of nomenclature, then sorted and classified in lipid classes. The amount of each lipid molecular species present in the sample was computed by use of AMDIS. The quantity of a particular molecular species was determined with this program by integration and calculation of the area under the gas chromatography (GC) total ion current peak associated with each lipid. Lipid molecular species concentrations were calculated relative to the internal standard by dividing of the area of each identified peak by the peak area of the internal standard. Processed data were subjected to statistical analysis (the term “significant” is used only when p<0.05 according to the t-test embedded in Excel.).

### Principal Components Analysis and Finding Potential Biomarkers

We identified a total of 106 lipid compounds in 22 allergenic pollens by using GC-MS analysis. We conducted a PubMed medical literature database search using the keyword phrases ‘“name of the identified pollen lipid compound” AND “allergy” OR “allergic rhinitis” OR “allergic asthma” OR “asthma” OR “inflammation” to explore the relationship between pollen lipid compounds and allergic diseases. No date restrictions were used. Only English language literature was searched. We reviewed the abstracts of the articles to identify lipid compounds that have been previously implicated in allergy, allergic rhinitis, allergic asthma, or inflammation. The relevant articles were then reviewed for specific data on lipid compounds related to allergy or allergic asthma. The strategy of this study was to focus on lipids that were found in most allergenic pollen. We selected ∼ 36 compounds for further analysis.

### Activation of Natural Killer T (**NKT)** Cells by Lipid Compounds

Vα14i NKT cell hybridomas (CD1d-restricted) have been described previously by Dr. A. Bendelac [31] (University of Chicago, Chicago, IL) and widely used for defining the dendritic cells (DCs) and the NKT cell-specific signaling pathways. Bone marrow-derived mouse DCs were prepared as described [32]. Myeloid-differentiation primary-response-gene 88 knock-out (MyD88^−/−^) mice were kindly provided by Drs. Kasper Hoebe and Bruce Beutler. We used the DCs/NKT co-culture to investigate the mechanism underlying the influence of individual pollen lipids namely, fatty acids (FAs), n-alkanes, sterols, and fatty alcohols on immune functions. DCs were harvested after 6 days of culture in RPMI 1640 medium (Invitrogen) supplemented with glutamine, antibiotics, 5×10^−5^ M 2-ME and 10% FCS with 2 ng/mL rm-GM-CSF (R&D systems). In these experiments, WT.B6 or MyD88^−/−^ DCs (1×10^5^) were plated and pulsed with either FAs and n-alkanes (1 µg/ml) or fatty alcohol, sterols, or other lipids (5 µg/ml) in 200 µl of complete medium in 96-well U-bottomed plates and kept in complete medium for 12 hours. DCs were washed once to remove lipids bound non-specifically before the NKT cell hybridomas (1×10^5^/well) were added to test for pollen-derived lipids reactivity. DC/NKT co-cultures were incubated for an additional 36 h at 37°C, with 5% CO2. CD1d cell surface expression by DCs was monitored in an experiment using flow cytometry.

### Determination of Cytokines in Cell Culture Supernatants

Cell-free culture supernatants were assayed 48 hours later for TNF-α, IL-2, IL-13, and IL-10 release by sandwich ELISA (eBiosciences, San Diego, USA) according to the manufacturer’s protocol.

### FACS and Intracellular Cytokine Staining (ICS)

Unseparated DC populations or purified cell populations were cultured in RPMI 1640 complete medium supplemented with 10% (v/v) FBS, 2.0 mM L-glutamine, 50 mM 2-mercaptoethanol, 100 U/ml penicillin and 100 mg/ml streptomycin at a density of 10^6^ cells/ml in (6 ml/well) in 6-well culture plates (Falcon, Lincoln Park, NJ) at 37°C in a 5% CO_2_ atmosphere. Cells were stimulated with 1 µg/ml lipid molecules for 24 to 36 hours in plates. After treatment with 10 µg/mL GolgiPlug, a protein transport inhibitor containing brefeldin A (eBiosciences) during the final 6 to 12 hours of stimulation, the cells were stained for surface markers with anti-CD3 APC-Cy7 (1 ug/10^6^ cells) and anti-CD4 Cy-Chrome (1 ug/10^6^ cells), fixed with Cytofix (eBiosciences) for 20 minutes at 4°C, and incubated with allophycocyanin (APC)-conjugated rat anti-mouse TNF-α (eBiosciences) in permeabilization buffer (PBS, 0.5% saponin, 2% fetal calf serum). Samples were stored at 4°C until flow cytometric analysis within 24 h.

### Endotoxin Determination by Limulus Amebocyte Lysate (LAL) Assay

We checked for the presence of endotoxin (lipopolysaccharide, LPS) as contaminant in lipid compounds to ensure that no contaminating molecules were responsible for the stimulation. Endotoxin determination in the lipids was carried out by LAL Pyrogent Plus gel clot assay as per the manufacture’s protocols (Cambrex Bio Science, Walkersville, MD, USA).

## Results

### GC-MS Profiling and Quantitation of Allergenic Pollen Lipids

We selected 22 known allergenic plant species for pollen lipid profiling, including 6 grasses, 2 weeds, and 14 trees. This selection was based on a rating of 7 or higher (10 being the most potent) on the OPALSTM (Ogren Plant Allergy Scale) [33], indicating a high level of allergenicity. To that end, three independent lipid extractions [n = 22 (species) ×3 (lipid extractions)] were extracted by use of chloroform, subjected to pre-column derivatization. Samples were subjected to GC-MS, and the area data for the fragment peak were recorded. We divided the area of each identified peak by the peak area of the internal standard to determine the amounts of individual compounds in each sample. Mass-spectral data were used for determination of the identity of each peak. The total ion chromatograms (TICs) were obtained for all pollen, followed by detailed data mining by use of extracted ion chromatograms (EICs) and statistical analysis.

We built a database library of the pollen lipids by matching acquired pollen-lipid mass spectra and retention times with the NIST/EPA/NIH mass-spectral library. Taking all of our data into account, we detected, identified, and quantitated more than 106 lipid molecular species, including FAs, n-alkanes, alcohols, sterols, and lipophilic vitamins.

**Figure 1:**
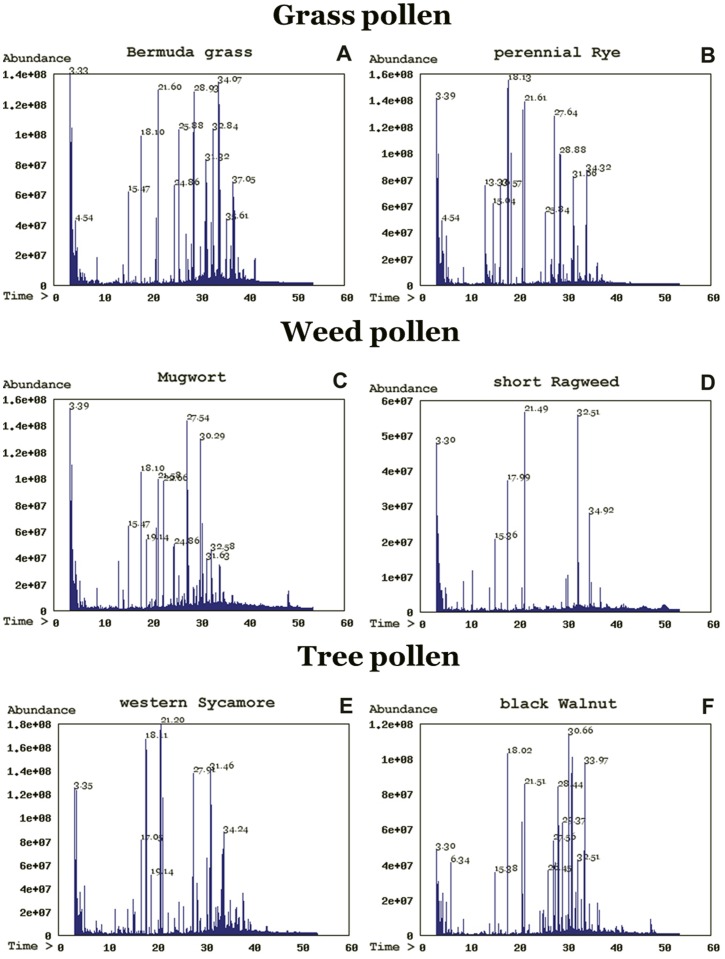
A typical total ion chromatogram (TIC) of trimethylsilyl (TMS)-derivatives of lipid molecular species obtained after separation of lipids extracted from six species of grass, weed, and tree pollen. The conditions for lipid analysis were as described in Methods.

Figure 1 presents an example gas chromatogram of the TIC of chloroform extract prepared from the 22 pollen species. The lipid profiling of the 22 species is shown in Tables 1,2,3,4,5,6,7,8,9,10,11,12, with the complete datasets of these species available as supplemental supporting information (Table S1, Table S2, Table S3 & Table S4). The values are the mean ± SEM of three independent samples.

**Table 1:** Distribution of Straight Chain Fatty Acids Identified and Quantitated by GC-MS in Pollen of 22 Species.

Assignment^a^	C5∶0	C7∶0	C9∶0	C10∶0	C12∶0	C14∶0	C15∶0	C16∶0
**Occurrence** b	**6**	**4**	**19**	**9**	**13**	**21**	**14**	**22**
**Retention Time (min)** c	**08.54±0.02**	**04.58±0.01**	**06.38±0.02**	**07.62±0.02**	**10.75±0.02**	**14.40±0.03**	**29.48±0.34**	**17.88±0.34**
**Pollen Grains**	**Peak Area Ratios, compound/IS** d							
**Bermuda Grass**			**0.083±0.01**		0.003±0.00	**0.194±0.01**	**0.020±0.01**	**0.545±0.75**
**Kentucky Blue Grass**					0.005±0.00	**0.254±0.01**	**0.022±0.01**	**0.003±0.00**
**Johnson Grass**	**0.005±0.01**		**0.036±0.01**	**0.008±0.00**		**0.203±0.01**	**0.024±0.00**	**0.002±0.00**
**Orchard Grass**			**0.079±0.01**		**0.010±0.00**	**0.621±0.12**		**6.914±1.45**
**Perennial Rye**	0.013±0.00		0.013±0.00	0.004±0.00	0.004±0.00	0.465±0.15	**0.056±0.03**	**6.457±5.53**
**Timothy Grass**						**0.503±0.13**	**0.044±0.02**	**8.422±2.11**
**Common Mugwort**			**0.109±0.01**	**0.016±0.00**	**0.038±0.03**	**0.270±0.07**	**0.050±0.00**	**0.003±0.00**
**Short Ragweed**			**0.006±0.00**	**0.091±0.01**	**0.497±0.05**	**0.296±0.04**	**0.023±0.01**	**1.892±0.25**
**European Alder**						**3.229±1.72**		**63.18±27.9**
**White Ash**			**0.065±0.00**			**0.209±0.01**		**1.707±0.18**
**White Birch**	0.010±0.00		**0.140±0.00**	**0.023±0.00**		**0.191±0.01**		**2.046±0.04**
**Box Elder**			**1.956±0.07**	0.060±0.00	**0.146±0.02**	**1.084±0.02**	**0.229±0.00**	**22.03±1.46**
**Mountain Cedar**			0.062±0.00	0.050±0.01	**0.189±0.08**	**0.480±0.13**		**4.614±0.07**
**Eastern Cottonwood**			0.027±0.01			**0.344±0.03**		**3.252±2.28**
**American Elm**			**0.130±0.02**			**0.380±0.10**	0.082±0.01	**8.736±1.58**
**Red Mulberry**			**3.029±0.33**					**45.96±2.01**
**Olive**		0.062±0.00	**0.525±0.03**	**0.022±0.01**	**0.792±0.53**	**1.832±0.27**	**0.058±0.00**	**0.282±0.23**
**Pecan**			**1.052±0.07**	0.004±0.00	**0.005±0.00**	**0.199±0.01**	**0.021±0.01**	**0.003±0.00**
**Red Oak**	0.008±0.00	**0.108±0.01**	**0.515±0.06**			**0.195±0.02**	**0.030±0.00**	**1.138±0.80**
**White Oak**	0.008±0.00	**0.155±0.03**	**0.638±0.04**	0.003±0.00	0.003±0.00	**0.149±0.00**	**0.025±0.00**	**0.613±0.87**
**Western Sycamore**	**0.008±0.00**		**0.027±0.01**			**0.247±0.06**	**0.051±0.00**	6.242±0.17
**Black Walnut**		**0.330±0.00**	**0.749±0.01**		**0.004±0.00**	**0.154±0.01**		**3.362±0.29**

Only compounds identified in the GC-MS relative quantitative analysis are shown. Compounds are sorted first by carbon-chain length, then by elution time in the gas chromatography and by abundance among the 22 pollen species.

a Identification of compounds was based on a match of the mass spectrum from the NIST/EPA/NIH library and on similar retention time.

b Indicates the number of pollen species in which the lipid molecular species was present.

c Means of retention times in minutes.

d Based on the relative peak area. The average concentration (mean ± SEM of 3 determinations) is given for samples. Averages of compounds identified in all three independent determinations are shown in bold. Average of compounds identified in only two out of three independent determinations are shown underlined.

n-nonadecane (nC19) TMS was used as internal standard (IS, retention time = 15.4 min) using a DB-5 60 column.

**Table 2:** Distribution of Straight Chain Fatty Acids Identified and Quantitated by GC-MS in Pollen of 22 Species.

Assignment^a^	C17∶0	C18∶0	C19∶0	C20∶0	C22∶0	C23∶0	C24∶0	C26∶0
**Occurrence^b^**	**20**	**6**	**17**	**21**	**21**	**14**	**3**	**4**
**Retention Time (min)^c^**	**19.76±0.17**	**21.54±0.03**	**23.26±0.07**	**24.82±0.03**	**27.88±0.04**	**16.18±0.18**	**30.71±0.04**	**32.14±0.02**
**Pollen Grains**	**Peak Area Ratios, compound/IS^d^**							
**Bermuda Grass**	**0.063±0.01**		**0.029±0.00**	**1.228±0.07**	**0.138±0.01**			
**Kentucky Blue Grass**	**0.116±0.00**		**0.041±0.01**	**0.203±0.00**	**0.058±0.00**	**0.002±0.00**		
**Johnson Grass**	**0.062±0.00**		**0.055±0.00**	**1.021±0.07**	**0.166±0.01**			
**Orchard Grass**	**0.215±0.13**		**0.384±0.04**	**3.378±0.38**	**0.416±0.03**	0.003±0.00	**0.368±0.02**	
**Perennial Rye**	**0.242±0.11**		**0.051±0.03**	**0.416±0.18**	**0.118±0.06**			
**Timothy Grass**	**0.202±0.04**		**0.148±0.05**	**1.690±0.41**	**0.415±0.04**			
**Common Mugwort**	**0.085±0.01**		**0.008±0.00**		**0.491±0.04**	**0.030±0.00**		
**Short Ragweed**	**0.081±0.01**	**3.055±0.40**		**0.035±0.01**	**0.030±0.02**		0.012±0.01	
**European Alder**		**60.48±29.08**	**2.858±1.28**	**6.277±2.81**	**31.54±14.5**	**6.620±3.40**		
**White Ash**	**0.062±0.02**			**0.235±0.01**	**0.157±0.02**	**0.006±0.00**		
**White Birch**	**0.066±0.01**	**2.323±0.17**	**0.198±0.01**	**0.309±0.02**	**1.751±0.07**			
**Box Elder**	**0.313±0.08**		**0.082±0.00**	**3.628±0.01**	**7.237±0.13**		**5.511±0.22**	
**Mountain Cedar**	**0.108±0.05**		6.563±1.21	**1.057±0.41**	**0.410±0.21**	**4.457±3.31**		
**Eastern Cottonwood**	**0.150±0.02**			**0.542±0.06**	**0.335±0.03**			**0.064±0.04**
**American Elm**	**0.113±0.03**	**4.819±0.80**		**0.517±0.05**	**0.402±0.02**	**0.161±0.03**		
**Red Mulberry**		**26.12±1.84**		1.188±0.00		**11.48±1.88**		**8.931±0.59**
**Olive**	**0.069±0.01**		0.007±0.00	**0.277±0.01**	**0.199±0.00**	**0.008±0.00**		
**Pecan**	**0.091±0.00**		**0.024±0.01**	**1.377±0.16**	**2.752±0.12**	**0.175±0.01**		0.071±0.00
**Red Oak**	**0.036±0.03**		**0.006±0.00**	**0.123±0.00**	**0.148±0.01**	**0.624±0.04**		
**White Oak**	**0.011±0.01**		0.003±0.00	**0.102±0.00**	**0.100±0.01**	**0.375±0.03**		0.057±0.02
**Western Sycamore**	**0.103±0.00**		**0.010±0.00**	**0.946±0.06**	2.290±2.28	**0.033±0.00**		
**Black Walnut**	**0.074±0.01**	**2.831±0.16**	**0.006±0.00**	**0.295±0.01**	**1.010±0.07**	**0.834±0.15**		

a, b, c, and **^d^** are described in the legend for Table 1.

**Table 3:** Distribution of Unsaturated Fatty Acids Identified and Quantitated by GC-MS in Pollen of 22 Species.

Assignment^a^	2-Propenoic acid C3∶1	3-Butenoic acid C4∶1	trans-9-hexadecenoic acid (palmitelaidic)	cis-11-octadecenoic (cis-vaccenic)	trans-11-octadecenoic (vaccenic)	9,12-octadecadienoic C18∶2 (n-6)	6,9,12-octadecatrienoic C18∶3 (n-6)	cis-11-eicosenoic C20∶1 (n-9)
**Occurrence^b^**	**11**	**2**	**9**	**18**	**11**	**22**	**12**	**8**
**Retention Time (min)^c^**	**04.10±0.22**	**04.96±0.12**	**17.68±0.035**	**21.18±0.067**	**21.20±0.05**	**21.02±0.035**	**21.16±0.041**	**24.43±0.028**
**Pollen Grains**	**Peak Area Ratios, compound/IS^d^**							
**Bermuda Grass**				0.006±0.00	**0.014±0.01**	**0.296±0.03**	**0.799±0.07**	
**Kentucky Blue Grass**				**0.022±0.00**		**0.145±0.00**	**2.245±0.03**	**0.024±0.00**
**Johnson Grass**	**0.003±0.00**		**0.005±0.00**	0.033±0.00		**0.457±0.04**	**1.623±0.13**	
**Orchard Grass**	**0.013±0.00**	**0.277±0.03**	**0.029±0.01**	**0.198±0.03**		**1.348±0.53**	**9.385±1.42**	
**Perennial Rye**			**0.021±0.01**	0.021±0.00	0.037±0.00	**0.671±0.25**	**7.634±3.04**	
**Timothy Grass**			**0.059±0.01**	0.135±0.03	0.160±0.01	**0.944±0.40**	**12.58±3.07**	
**Common Mugwort**	**0.006±0.00**		**0.016±0.00**	**1.024±0.05**	0.558±0.52	**0.102±0.01**		
**Short Ragweed**				0.071±0.02		**0.274±0.03**		
**European Alder**					**11.22±9.83**	**67.01±29.6**	**39.71±16.52**	
**White Ash**				**2.133±0.44**	**0.682±0.73**	**0.994±0.03**		**0.846±0.09**
**White Birch**	**0.007±0.00**	0.014±0.00		**0.421±0.02**		**0.685±0.03**		**0.023±0.00**
**Box Elder**	**0.107±0.04**		**0.103±0.01**	**2.630±3.45**	4.092±3.70	**9.859±0.24**	**24.34±1.24**	**1.511±0.06**
**Mountain Cedar**				**3.071±0.40**		**1.864±0.37**		
**Eastern Cottonwood**					**4.362±0.21**	**4.428±0.55**	**9.086±0.33**	**0.574±0.01**
**American Elm**	0.068±0.00				0.141±0.06	**6.013±0.67**	**13.48±2.03**	
**Red Mulberry**						4.904±0.00		
**Olive**	**0.005±0.00**		**0.076±0.00**	**1.150±1.32**	3.058±0.18	**3.302±0.06**		**0.041±0.00**
**Pecan**			**0.048±0.00**	5.041±0.04		**6.174±0.36**		**0.021±0.00**
**Red Oak**	**0.009±0.00**			**0.398±0.13**		**0.083±0.02**		0.077±0.04
**White Oak**	0.004±0.00			**0.267±0.00**		**0.462±0.01**		
**Western Sycamore**	**0.016±0.01**		**0.071±0.01**	0.214±0.00	**1.167±0.44**	**7.992±0.84**	**6.338±0.37**	
**Black Walnut**	0.006±0.00			**0.034±0.00**		**1.047±0.03**	**1.888±0.05**	

a, b, c, and **^d^** are described in the legend for Table 1.

**Table 4:** Distribution of Dicarboxylic Acids Identified and Quantitated by GC-MS in Pollen of 22 Species.

Assignment^a^	cis-2-butenedioic acid (maleic)	trans-2-Butenedioic acid (fumaric)	Butanedioic acid (succinic)	Pentanedioic acid (glutaric)	Tetrahydroxyhexanedioic acid (glucaric)	Tetrahydroxyadipic acid (mucic or galactaric)	Octanedioic acid (suberic)	benzene-1,2-dicarboxylic acid (phthalic)	1,9-Nonanedicarboxylic acid (undecanedioic)
**Occurrence^b^**	**18**	**11**	**16**	**3**	**4**	**3**	**2**	**7**	**4**
**Retention Time (min)^c^**	**07.88±0.02**	**06.30±0.06**	**05.81±0.01**	**09.77±0.03**	**17.250±0.28**	**17.50±0.01**	**11.53±0.09**	**26.32±0.03**	**17.00±0.01**
**Pollen Grains**	**Peak Area Ratios, compound/IS^d^**								
**Bermuda Grass**	**0.042±0.04**			0.008±0.00					
**Kentucky Blue Grass**	**0.129±0.06**	0.009±0.00	**0.014±0.00**		0.009±0.00				
**Johnson Grass**				**0.009±0.00**					
**Orchard Grass**	**0.061±0.01**			**0.019±0.01**					
**Perennial Rye**	**0.095±0.11**		**0.043±0.02**					**0.020±0.01**	
**Timothy Grass**	**1.311±0.56**		**0.229±0.08**						
**Common Mugwort**		**0.005±0.00**	**0.010±0.00**				**0.030±0.00**	**0.059±0.01**	**0.014±0.01**
**Short Ragweed**		**0.001±0.00**	**0.022±0.01**						
**European Alder**	**23.02±8.58**				**1.561±0.79**				
**White Ash**	**0.060±0.02**	0.017±0.00	**0.007±0.00**						
**White Birch**	**0.178±0.06**	**0.026±0.00**	**0.040±0.01**					**0.024±0.00**	
**Box Elder**	**1.898±0.44**	**0.077±0.02**	**0.236±0.05**						
**Mountain Cedar**									
**Eastern Cottonwood**	**1.321±0.64**	**0.051±0.03**	**0.136±0.08**		0.037±0.01	0.036±0.02			
**American Elm**	**1.253±0.67**	0.065±0.03				**0.073±0.06**		0.024±0.00	
**Red Mulberry**	**64.46±9.93**	**2.040±1.07**	**5.328±0.89**		3.172±0.00	**2.421±1.33**			
**Olive**	**0.120±0.08**		**0.014±0.01**					**0.067±0.00**	0.029±0.01
**Pecan**	**0.030±0.01**		**0.005±0.00**				**0.010±0.00**		
**Red Oak**	**0.144±0.04**	0.004±0.00	0.009±0.00						**0.015±0.01**
**White Oak**	**0.105±0.02**		0.009±0.00						**0.016±0.01**
**Western Sycamore**	**0.289±0.05**	0.025±0.01	**0.028±0.00**					**0.008±0.00**	
**Black Walnut**	**0.081±0.01**		**0.016±0.00**					**0.031±0.00**	

a, b, c, and **^d^** are described in the legend for Table 1.

**Table 5:** Distribution of Dicarboxylic Acids Identified and Quantitated by GC-MS in Pollen of 22 Species.

Assignment^a^	2-hydroxypropane-1,2,3-tricarboxylic acid (citric)	1,2,3-Propanetricarboxylic acid (isocitric)	2,3,4,5,6-pentahydroxyhexanoic acid	3-(4-hydroxy-3-methoxy-phenyl)prop-2-enoic) (cinnammic)	4-Hydroxyanthraquinone-2-carboxylic acid	2,4,6-Trihydroxybenzoic acid	2,3,4-Trihydroxybutyric acid	2-Piperidinecarboxylic acid
**Occurrence^b^**	**17**	**5**	**8**	**7**	**4**	**2**	**2**	**4**
**Retention Time (min)^c^**	**13.64±0.02**	**13.66±0.01**	**16.03±1.03**	**18.88±0.09**	**17.00±0.01**	**16.63±0.00**	**08.92±0.08**	**06.51±0.01**
**Pollen Grains**	**Peak Area Ratios, compound/IS^d^**							
**Bermuda Grass**	0.007±0.00	**0.031±0.03**	0.012±0.00	0.002±0.00				
**Kentucky Blue Grass**	**0.018±0.01**	0.020±0.01						
**Johnson Grass**	**0.006±0.00**		**0.018±0.00**	**0.022±0.00**				
**Orchard Grass**		**0.155±0.08**	**0.026±0.01**					
**Perennial Rye**	0.012±0.00	**0.318±0.44**	**0.008±0.00**					
**Timothy Grass**		**15.03±6.40**						
**Common Mugwort**	**0.005±0.00**							
**Short Ragweed**	0.003±0.00							
**European Alder**	**50.85±16.4**			**0.458±0.22**			**9.298±3.48**	**29.84±9.92**
**White Ash**	0.013±0.00							
**White Birch**						**0.056±0.00**		
**Box Elder**	**0.353±0.12**							
**Mountain Cedar**				0.076±0.08	**0.155±0.05**			
**Eastern Cottonwood**	**1.590±0.26**		**0.052±0.02**	0.085±0.00		**0.545±0.14**		
**American Elm**	**0.659±0.34**		0.630±0.32	**0.044±0.02**				0.583±0.30
**Red Mulberry**	**38.22±20.13**						**6.479±3.75**	**8.258±3.79**
**Olive**	**0.038±0.03**		**0.070±0.04**					
**Pecan**			**0.057±0.06**		**0.051±0.01**			
**Red Oak**	**0.015±0.00**				**0.023±0.01**			
**White Oak**	0.006±0.00				**0.008±0.00**			
**Western Sycamore**	**0.141±0.01**			0.013±0.00				**0.033±0.00**
**Black Walnut**	**0.013±0.00**							

a, b, c, and **^d^** are described in the legend for Table 1.

**Table 6:** Distribution of n-Alkanes (Saturated Hydrocarbons) Identified and Quantitated by GC-MS in Pollen of 22 Species.

Assignment^a^	nC6	nC10	nC12	nC14	nC16	nC17	nC21	nC23	nC24	nC25
**Occurrence^b^**	**19**	**11**	**10**	**12**	**16**	**13**	**12**	**16**	**10**	**12**
**Retention Time (min)^c^**	**07.36±0.57**	**07.86±0.18**	**05.24±0.01**	**07.29±1.51**	**20.75±1.056**	**12.54±2.76**	**19.12±0.02**	**22.59±0.02**	**24.24±0.01**	**30.25±0.01**
**Pollen Grains**	**Peak Area Ratios, compound/IS^d^**									
**Bermuda Grass**	**0.034±0.00**	**0.026±0.00**	**0.009±0.00**	**0.024±0.01**	**0.019±0.00**	0.012±0.00		**0.048±0.00**	**0.079±0.01**	**2.609±0.17**
**Kentucky Blue Grass**	**0.047±0.00**	**0.025±0.00**	**0.016±0.00**	**0.024±0.00**	**0.127±0.01**	**0.010±0.00**	**0.008±0.00**	**0.028±0.00**		
**Johnson Grass**	**0.031±0.00**	**0.010±0.00**		**0.016±0.00**	**0.009±0.00**	**0.014±0.00**	**0.012±0.00**	**0.111±0.00**	**0.123±0.01**	**1.669±0.07**
**Orchard Grass**	0.091±0.01	0.042±0.01	**0.035±0.00**	**0.047±0.01**	**0.022±0.01**	**0.050±0.01**	**0.109±0.02**	**1.889±0.15**	**0.343±0.15**	**6.981±0.59**
**Perennial Rye**	**0.066±0.02**	0.020±0.01	0.012±0.00	0.014±0.00	**0.034±0.01**	0.031±0.00		**0.045±0.01**	0.039±0.01	**3.059±1.24**
**Timothy Grass**	**0.076±0.02**							**0.790±0.07**	0.080±0.01	**5.637±1.04**
**Common Mugwort**	**0.042±0.00**		0.005±0.00	0.013±0.00	0.007±0.00	**0.013±0.00**	**1.053±0.07**	**2.025±0.21**	**0.082±0.01**	**0.434±0.02**
**Short Ragweed**	0.013±0.00	**0.010±0.00**		**0.009±0.00**	**0.007±0.00**					
**European Alder**										
**White Ash**	**0.040±0.00**	**0.023±0.01**	**0.017±0.01**	**0.022±0.01**	0.019±0.00	0.011±0.00	**0.037±0.01**	**1.089±0.05**	**0.293±0.02**	**2.567±0.30**
**White Birch**	**0.036±0.00**		**0.014±0.00**		**0.989±0.03**	**0.003±0.00**				
**Box Elder**	**0.067±0.03**	0.075±0.00				**0.121±0.01**	**0.123±0.02**	**2.208±0.13**	0.428±0.01	**15.60±1.60**
**Mountain Cedar**	**0.066±0.02**				3.790±0.89					
**Eastern Cottonwood**	**0.053±0.03**				0.053±0.03			**0.600±0.09**		4.728±0.41
**American Elm**								**0.343±0.05**	0.252±0.02	0.446±0.08
**Red Mulberry**										
**Olive**	**0.026±0.00**	**0.012±0.01**	**0.011±0.00**	**0.018±0.00**	**0.009±0.00**	**0.042±0.00**	**0.296±0.03**	**0.341±0.02**		**0.395±0.01**
**Pecan**	**0.029±0.00**	**0.020±0.00**	**0.013±0.00**	**0.015±0.00**	**0.066±0.00**	**0.013±0.00**	**0.017±0.00**	**0.024±0.00**		
**Red Oak**	**0.037±0.01**				**0.199±0.01**		**0.026±0.00**	**0.083±0.01**		
**White Oak**	**0.031±0.00**			**0.012±0.00**	0.084±0.01	0.011±0.00	0.025±0.00	**0.078±0.00**		
**Western Sycamore**	**0.033±0.00**						**1.832±0.11**	**0.630±0.01**	**0.128±0.04**	**0.857±0.03**
**Black Walnut**	0.007±0.00	**0.013±0.00**	**0.004±0.00**	**0.013±0.00**	**0.636±0.00**	0.009±0.00	0.025±0.00			

a, b, c, and **^d^** are described in the legend for Table 1.

**Table 7:** Distribution of n-Alkanes (Saturated Hydrocarbons) Identified and Quantitated by GC-MS in Pollen of 22 Species.

Assignment^a^	nC26	nC27	nC28	nC29	nC30	nC31	nC32	nC33	nC35
**Occurrence^b^**	**9**	**18**	**10**	**18**	**10**	**10**	**3**	**5**	**3**
**Retention Time (min)^c^**	**27.36±0.01**	**28.85±0.05**	**25.86±0.03**	**31.63±0.03**	**32.96±0.01**	**34.29±0.02**	**29.31±0.46**	**37.38±0.02**	**41.50±0.01**
**Pollen Grains**	**Peak Area Ratios, compound/IS^d^**								
**Bermuda Grass**	**0.627±0.06**	**4.388±1.04**	**0.445±0.05**	**1.268±0.07**	0.104±0.01	**1.275±0.15**	**0.052±0.01**	**1.326±0.16**	**0.281±0.06**
**Kentucky Blue Grass**		**0.995±0.03**	**0.052±0.01**	**1.427±0.02**	**0.033±0.00**	**0.520±0.01**			
**Johnson Grass**	**0.539±0.04**	**4.649±0.40**	**0.458±0.03**	**1.012±0.08**	**0.127±0.01**	**0.719±0.04**		**0.694±0.03**	**0.383±0.03**
**Orchard Grass**		**9.032±0.82**	**0.702±0.14**	**7.416±1.85**	**0.725±0.13**	**5.751±0.51**			
**Perennial Rye**	**0.258±0.10**	**5.495±2.22**	**0.299±0.12**	**3.809±1.82**	**0.283±0.13**	**4.794±2.15**	**0.143±0.07**		
**Timothy Grass**	**0.486±0.14**	**11.72±3.31**	**0.396±0.13**	**5.884±2.03**	**0.351±0.10**	**5.928±2.02**			
**Common Mugwort**		**0.221±0.01**		**0.672±0.05**	**0.042±0.00**	**0.590±0.04**			
**Short Ragweed**									
**European Alder**									
**White Ash**	**0.164±0.01**	**1.493±0.14**	**0.043±0.00**	**0.259±0.01**		**0.082±0.01**			
**White Birch**		**1.422±0.04**		**0.252±0.02**					
**Box Elder**		**27.52±0.64**		**10.98±0.74**	**0.680±0.14**	**11.38±0.48**		0.970±0.00	
**Mountain Cedar**								0.816±0.04	1.376±0.03
**Eastern Cottonwood**	**0.469±0.06**	**8.430±0.69**		**3.583±0.39**					
**American Elm**		**0.599±0.13**	**0.268±0.13**	**2.923±0.43**	0.217±0.02				
**Red Mulberry**		**6.363±1.51**		**14.62±5.36**					
**Olive**	**0.025±0.00**	**0.367±0.00**	**0.062±0.01**	**0.906±0.01**	**0.104±0.00**	**1.022±0.06**	**0.095±0.02**	**0.608±0.03**	
**Pecan**		**0.065±0.01**		**0.079±0.01**					
**Red Oak**		**0.113±0.01**		**0.144±0.14**					
**White Oak**		**0.079±0.00**		**0.026±0.03**					
**Western Sycamore**	**0.133±0.05**	**1.056±0.03**	**0.104±0.05**	**0.559±0.03**					
**Black Walnut**									**0.028±0.08**

a, b, c, and **^d^** are described in the legend for Table 1.

**Table 8:** Distribution of Fatty Alcohols, Alkenes, Mono-unsaturated Alcohols, and Aldehydes Identified and Quantitated by GC-MS in Pollen of 22 Species.

Assignment^a^	1-Decanol (C10)	2-Hexyl-1-octanol (C14)	1-Nonadecanol (C19)	1-Pentacosanol (C25)	1-Heptacosanol (C27)	Anteiso-heptacosanol	1- Octacosanol (C28)	1-Triacontanol (C30)	4-Hydroxyphenylethanol
**Occurrence^b^**	**5**	**13**	**1**	**2**	**7**	**6**	**16**	**3**	**4**
**Retention Time (min)^c^**	**05.99±0.05**	**08.83±0.15**	**28.45±0.05**	**25.44±0.09**	**28.54±0.07**	**33.99±1.29**	**34.76±0.03**	**32.74±0.08**	**09.40±0.02**
**Pollen Grains**	**Peak Area Ratios, compound/IS^d^**								
**Bermuda Grass**	0.041±0.01	**0.030±0.01**			**0.535±0.06**		**0.027±0.00**	**1.788±0.21**	
**Kentucky Blue Grass**		**0.024±0.00**					**0.017±0.01**		
**Johnson Grass**		0.018±0.00			**0.781±0.04**		**0.031±0.01**		
**Orchard Grass**		**0.059±0.01**		2.057±1.31	**2.721±0.29**		0.006±0.00		
**Perennial Rye**		0.033±0.00			0.155±0.01				
**Timothy Grass**					**8.158±3.94**				
**Common Mugwort**		**0.021±0.00**					**0.019±0.00**		
**Short Ragweed**		**0.019±0.01**							
**European Alder**							**0.470±0.22**		
**White Ash**	**0.061±0.00**	0.038±0.01						**0.331±0.04**	**0.059±0.00**
**White Birch**	**0.041±0.00**	**0.016±0.00**	**0.452±0.05**			**0.026±0.01**	0.668±0.15		**0.041±0.01**
**Box Elder**		0.072±0.03				**0.075±0.00**	**2.729±0.19**		
**Mountain Cedar**						**0.058±0.03**	**1.739±0.54**		
**Eastern Cottonwood**							**0.194±0.01**		
**American Elm**					**8.904±5.53**		**0.028±0.02**	**0.592±0.10**	
**Red Mulberry**							**1.452±0.14**		
**Olive**	0.039±0.00								**0.002±0.00**
**Pecan**	0.040±0.00						**0.033±0.00**		
**Red Oak**		**0.025±0.00**				**0.061±0.00**	**0.161±0.01**		
**White Oak**		**0.017±0.01**				**0.016±0.01**	**0.108±0.01**		
**Western Sycamore**						0.261±0.02			**0.032±0.00**
**Black Walnut**		**0.022±0.00**		**0.380±0.02**	**2.665±0.58**		**0.034±0.01**		

a, b, c, and **^d^** are described in the legend for Table 1.

**Table 9:** Distribution of Fatty Alcohols, Alkenes, Mono-unsaturated Alcohols, and Aldehydes Identified and Quantitated by GC-MS in Pollen of 22 Species.

Assignment^a^	1-Tetradecene (C14∶1)	cis-9-Tricosene (C23∶1)	Z-12-Pentacosene (C25∶1)	9-Hexacosene (C26∶1)	1,21-Docosadiene (C22∶2)	2-hexadecen-1-ol (C16∶1)	cis-9-Eicosen-1-ol (C20∶1)	Octadecanal	Nonanal
**Occurrence^b^**	**9**	**4**	**10**	**4**	**5**	**6**	**2**	**2**	**6**
**Retention Time (min)^c^**	**08.78±0.17**	**22.18±0.07**	**25.45±0.04**	**27.03±0.10**	**24.74±0.03**	**14.28±0.11**	**21.34±0.05**	**17.70±0.04**	**04.26±0.01**
**Pollen Grains**	**Peak Area Ratios, compound/IS^d^**								
**Bermuda Grass**	**0.029±0.00**								
**Kentucky Blue Grass**	0.022±0.01		**0.019±0.00**						
**Johnson Grass**	0.021±0.00		**0.069±0.01**	0.013±0.00	0.020±0.00				
**Orchard Grass**	**0.043±0.01**		**3.526±0.21**	**0.123±0.04**		**0.134±0.09**			
**Perennial Rye**	0.023±0.00		**0.069±0.00**						
**Timothy Grass**			**1.066±0.07**	0.077±0.00		**0.155±0.00**			
**Common Mugwort**	0.016±0.00								
**Short Ragweed**									
**European Alder**									
**White Ash**		**0.044±0.01**	**0.165±0.02**						
**White Birch**									
**Box Elder**		**0.208±0.02**			**0.462±0.06**	0.071±0.01		0.139±0.01	**0.260±0.04**
**Mountain Cedar**					0.225±0.03				
**Eastern Cottonwood**			0.322±0.04			0.093±0.01			
**American Elm**			**1.156±0.08**	**0.501±0.02**					
**Red Mulberry**									
**Olive**	0.017±0.00	**0.177±0.02**	**0.077±0.00**			**0.073±0.01**			**0.046±0.00**
**Pecan**	0.019±0.00				**0.514±0.01**		**0.244±0.09**		**0.214±0.03**
**Red Oak**	0.028±0.00								**0.054±0.01**
**White Oak**									**0.042±0.03**
**Western Sycamore**						**0.043±0.01**			
**Black Walnut**		**0.046±0.00**	0.352±0.01		**0.311±0.02**		**0.079±0.01**	**0.045±0.00**	**0.178±0.02**

a, b, c, and **^d^** are described in the legend for Table 1.

**Table 10:** Distribution of Sterols and Terpenes Identified and Quantitated by GC-MS in Pollen of 22 Species.

Assignment^a^	β-Sitosterol	[(3β-,24R)-ergost-5-en-3-yl]oxy]	Stigmastan-3,5-diene	α-Tocopherol (vitamin E)	Stigmasterol	Tocopherol-γ	β-Amyrin	Stigmasta-3,5-dien-7-one	Ergosta-5,24-dien-3-ol, acetate, (3-β.)-	3-β-ergost-8(14)-en-3-yl
**Occurrence^b^**	**16**	**13**	**12**	**9**	**6**	**5**	**5**	**3**	**2**	**1**
**Retention Time (min)^c^**	**38.12±0.05**	**36.64±0.02**	**34.43±.0.03**	**34.78±.0.03**	**37.05±0.03**	**32.79±0.08**	**38.90±0.33**	**39.85±0.06**	**33.17±0.02**	**37.73±0.01**
**Pollen Grains**	**Peak Area Ratios, compound/IS^d^**									
**Bermuda Grass**	**0.385±0.03**	**0.388±0.05**	**0.047±0.02**	**0.062±0.00**	**0.770±0.04**					
**Kentucky Blue Grass**	**0.053±0.00**	**0.081±0.02**			0.069±0.02				**0.028±0.02**	
**Johnson Grass**	**0.271±0.04**	**0.229±0.02**	**0.023±0.00**							**0.002±0.00**
**Orchard Grass**	**0.653±0.44**	**0.787±0.09**		**0.027±0.00**	**2.996±0.30**	0.011±0.00				
**Perennial Rye**		**0.008±0.00**			0.020±0.00				**0.100±0.06**	
**Timothy Grass**		**0.376±0.01**	**0.033±0.01**	**0.058±0.03**	**2.19±0.41**					
**Common Mugwort**				**0.007±0.00**			**0.032±0.01**			
**Short Ragweed**				0.003±0.00						
**European Alder**	22.46±3.53	2.466±0.37	**1.291±0.81**							
**White Ash**	**2.935±0.05**	**0.267±0.03**		**0.297±0.01**		**0.044±0.00**	0.058±0.00			
**White Birch**	**0.192±0.02**									
**Box Elder**	**6.068±0.66**		0.132±0.00		1.423±0.06	**0.420±0.04**	**7.175±0.15**			
**Mountain Cedar**	2.349±0.15	**0.312±0.17**								
**Eastern Cottonwood**	**2.858±0.45**		**0.141±0.02**	**0.892±0.09**		**0.035±0.00**	**0.483±0.05**			
**American Elm**	**1.808±0.15**	**0.017±0.00**	**0.140±0.03**							
**Red Mulberry**										
**Olive**	**1.330±0.03**	**0.062±0.02**		**0.081±0.01**		**0.012±0.00**	**0.212±0.01**			
**Pecan**	**0.615±0.03**	**0.022±0.00**	**0.053±0.01**					**0.022±0.01**		
**Red Oak**			**0.025±0.00**					**0.181±0.02**		
**White Oak**	**0.549±0.04**		**0.060±0.01**					0.038±0.00		
**Western Sycamore**	**1.796±0.02**	**0.126±0.01**	**0.047±0.01**	0.112±0.01						
**Black Walnut**	**0.073±0.00**		**0.009±0.00**							

a, b, c, and **^d^** are described in the legend for Table 1.

**Table 11:** Distribution of Other Lipid Molecular Species Identified and Quantitated by GC-MS in Pollen of 22 Species.

Assignment^a^	Benzoic acid	Glucitol	Benzene	Myo-Inositol	1,8-Dihydroxy-3-methylanthraquinone	Arabinitol	Phosphoric acid	(Z)-14-C29∶1	Pyridine
**Occurrence^b^**	**21**	**19**	**17**	**17**	**13**	**12**	**11**	**11**	**7**
**Retention Time (min)^c^**	**06.58±0.01**	**15.74±0.07**	**05.43±0.01**	**18.71±0.03**	**26.51±0.03**	**11.70±0.07**	**12.55±0.02**	**31.40±0.05**	**04.11±0.42**
**Pollen Grains**	**Peak Area Ratios, compound/IS^d^**								
**Bermuda Grass**	**0.046±0.00**	**0.005±0.00**	**0.092±0.04**	**0.507±0.63**		**0.006±0.00**		**0.864±0.16**	
**Kentucky Blue Grass**	**0.097±0.00**	0.004±0.00	**0.217±0.01**	**0.365±0.19**			**0.025±0.00**	**0.231±0.02**	**0.018±0.00**
**Johnson Grass**	**0.070±0.00**		**0.103±0.00**	**0.047±0.00**		0.005±0.00	**0.021±0.00**	**2.295** **±0.02**	
**Orchard Grass**	**0.181±0.03**		**0.329±0.05**	**1.530±0.75**			**0.042±0.03**	**7.864±0.85**	**0.691±0.21**
**Perennial Rye**	**0.137±0.04**	**0.012±0.01**	**0.166±0.01**	**2.673±2.57**				**0.836±0.28**	
**Timothy Grass**	**0.142±0.03**	**0.096±0.05**				**0.116±0.05**	**0.378±0.18**	**6.127±0.68**	
**Common Mugwort**	**0.073±0.01**	**0.008±0.00**	**0.047±0.01**		**0.008±0.00**				
**Short Ragweed**	**0.032±0.01**	**0.012±0.00**	0.016±0.01		0.008±0.00	**0.013±0.00**			**0.024±0.00**
**European Alder**	1.760±0.62	1.092±0.21		2.557±0.70	**5.127±2.37**		**16.92±1.68**		
**White Ash**	**0.113±0.00**	**0.241±0.12**	**0.211±0.07**	**0.024±0.01**	0.001±0.00			**0.922±0.06**	0.024±0.00
**White Birch**	0.102±0.00		**0.147±0.02**	0.022±0.01		**0.047±0.01**			**0.035±0.01**
**Box Elder**	**0.392±0.03**	**0.274±0.03**	**0.099±0.01**	**0.423±0.15**		**0.127±0.03**	**0.909±0.18**		**1.501±0.20**
**Mountain Cedar**	**0.261±0.04**	0.081±0.00			10.17±0.30				
**Eastern Cottonwood**	**0.156±0.01**	**0.908±0.41**	0.149±0.05	**0.225±0.20**			1.369±0.47	**3.868±0.33**	
**American Elm**	0.067±0.01	**0.471±0.25**		**1.106±0.54**	**0.274±0.04**	0.086±0.02	**0.219±0.21**	**8.038±0.72**	
**Red Mulberry**		1.877±0.00		**13.45±3.94**	**2.213±0.53**	0.955±0.00	5.530±0.00		
**Olive**	**0.075±0.00**	**0.075±0.06**	**0.136±0.02**	**0.063±0.04**	**0.003±0.00**				
**Pecan**	**0.102±0.00**	**0.102±0.09**	**0.103±0.03**		**0.614±0.04**	**0.009±0.00**		**1.821±0.07**	
**Red Oak**	**0.113±0.01**	**0.019±0.01**	**0.022±0.01**	**0.015±0.01**	**0.311±0.02**	**0.014±0.01**			
**White Oak**	**0.087±0.01**	**0.014±0.00**	**0.025±0.01**	**0.015±0.01**	**0.104±0.01**	**0.011±0.00**			
**Western Sycamore**	**0.086±0.00**	**0.660±0.00**	**0.124±0.02**	**0.215±0.05**	**0.167±0.01**	**0.026±0.00**	**0.054±0.03**		
**Black Walnut**	0.059±0.01	**0.002±0.00**	**0.023±0.01**	**0.012±0.00**	**1.035±0.01**		**0.038±0.01**	**4.281±0.16**	**0.025±0.00**

a, b, c, and **^d^** are described in the legend for Table 1A.

**Table 12:** Distribution of Other Lipid Molecular Species Identified and Quantitated by GC-MS in Pollen of 22 Species.

Assignment^a^	Phenol	Acetoacetic acid	Pentafluoropropionic acid	Benzaldehyde	Ribitol	L-Ascorbic acid	Inositol	Cinnamate	α-Tocopherolhydroquinone
**Occurrence^b^**	**6**	**6**	**5**	**5**	**4**	**4**	**3**	**2**	**2**
**Retention Time (min)^c^**	**06.81±0.01**	**05.01±0.14**	**12.83±0.11**	**06.81±0.01**	**16.07±0.03**	**16.07±0.03**	**17.52±0.01**	**19.67±0.01**	**36.99±0.01**
**Pollen Grains**	**Peak Area Ratios, compound/IS^d^**								
**Bermuda Grass**	**0.016±0.00**	**0.011±0.00**	**0.056±0.01**						
**Kentucky Blue Grass**		0.013±0.00	0.061±0.00	**0.010±0.00**					
**Johnson Grass**		**0.038±0.01**							
**Orchard Grass**		**0.236±0.02**	0.091±0.01						
**Perennial Rye**				0.004±0.00					
**Timothy Grass**		**0.046±0.02**				**0.055±0.02**	**0.120±0.05**		
**Common Mugwort**	0.016±0.00			**0.009±0.00**					
**Short Ragweed**	**0.027±0.01**								
**European Alder**				0.503±0.19					
**White Ash**			0.059±0.00		**0.140±0.07**				
**White Birch**	0.013±0.00	**0.012±0.01**							
**Box Elder**	0.009±0.00					**0.175±0.09**		**0.037±0.02**	
**Mountain Cedar**									
**Eastern Cottonwood**					**0.395±0.17**	0.068±0.03		**0.240±0.04**	**0.095±0.02**
**American Elm**									
**Red Mulberry**									
**Olive**					**0.036±0.01**				**0.041±0.01**
**Pecan**			**0.036±0.01**						
**Red Oak**							**0.013±0.00**		
**White Oak**							**0.014±0.00**		
**Western Sycamore**				**0.009±0.00**	**0.680±0.05**				
**Black Walnut**	**0.034±0.00**					**0.011±0.00**			

a, b, c, and **^d^** are described in the legend for Table 1.

### Straight-Chain Fatty Acids (SCFAs)

We identified 16 SCFAs in the lipid extracts of the 22 pollen species. Tables 1 & 2 show that SCFAs are widely distributed among the pollens, with C14-C22 predominating. GC-MS analysis detected a marked interspecies variation in the composition and concentration of SCFAs. The six most abundant SCFAs present in the pollen lipids were: Hexadecanoic acid (palmitic, 16∶0), tetradecanoic acid (myristic, 14∶0), eicosanoic acid (arachidic, 20∶0), docosanoic acid (behenic, 22∶0), heptadecanoic acid (margaric, 17∶0) and nonanoic acid (C9∶0). In addition, octadecanoic acid (stearic, C18∶0) was present in only six species. SCFAs, with chain lengths less than 10 and greater than 22, were not as predominant. Nonadecanoic acid (C19∶0), tricosanoic acid (C23∶0), pentadecanoic acid (C15∶0), and dodecanoic acid (C12∶0) were found in 17, 14, 14, and 13 of the 22 species, respectively, whereas heptanoic acid (C7∶0), hexacosanoic acid (C26∶0), and tetracosanoicoleic acid (C24∶0) were found in only 4, 4, and 3 species, respectively. European alder and red mulberry had the highest concentration of C16∶0 and C18∶0 FAs. The concentration of these two FAs far exceeded that found in the other 20 plant species. For some species, the following SCFAs: C16∶0, C23∶0, C12∶0, C19∶0, and C12∶0, were present at very low concentrations. The average peak-area ratio for the remainder of the FAs shown in Table 1 & 2 ranged from one to 10. Most species contained more SCFAs than unsaturated FAs.

### Unsaturated Fatty Acids (USFAs)

The presence and abundance of USFAs displayed marked variation among the different species (see Table 3). 9,12-octadecadienoic acid (linoleic, C18∶2), 11-cis-octadecenoic acid (cis-vaccenic, C18∶1) and 9,12,15-octadecatrienoic acid (C18∶3) were present in 22, 18, and 12 species, respectively. Linoleic acid was the dominant polyunsaturated fatty acid in the pollen lipids for all tested pollens. The peak-area ratio of 9,12,15-octadecatrienoic acid (C18∶3) in the species where it was detected was greater than that of the other USFAs.

### Dicarboxylic Acids

In contrast to the saturated FAs data, significant differences in the pattern of dicarboxylic acids were observed among the 22 species. The major FAs, in order of decreasing abundance among the 22 species, were (Z)-butenendioic, butanedioic, and buteneioic (E) acids (see Tables 4 & 5).

### n-Alkanes

Tables 6 & 7 summarize GC-MS data obtained from the analysis of the lipid of n-alkanes of the pollen. The composition of the 22 pollen varieties consisted of 25 different saturated, normal, and branched-chain hydrocarbons between C6–C35 and the straight chain; both odd and even carbon numbers were predominant in the mixture. Noteworthy was the identification of the odd-numbered carbon atom series C27, C29, C23, C17, and C21 as the most abundant n-alkanes. Long-chain n-alkanes C31∶0 were detected in all grasses, but in very few other samples. This was also true for C24∶0. Of particular interest was the large difference between the abundance of n-alkanes found in grasses compared to weed and tree pollen, as shown in Tables 6 & 7. A large difference was observed in the concentration of C25–C31, which were higher in grass pollen and tree pollen than in weed pollen. On the other hand, the concentration of C6–C21 was lower as compared to longer-chain hydrocarbons. In addition, no hydrocarbons were identified in red mulberry. GC-MS lipid profiling of pollen lipids indicated that aliphatic hydrocarbons (n-alkanes) were predominant constituents of lipids in all species, with some similarity among some tree pollens and grass pollens and differences among others. The most common saturated hydrocarbons were in the C6 to C14, C16 to C19, and C21 to C31 regions.

### Alkenes, Fatty Alcohols, Mono-unsaturated Alcohols, and Aldehydes

Tables 8 & 9 show compounds obtained from the analysis of pollen lipids categorized into four classes: alkenes, fatty alcohols, mono-unsaturated alcohols, and aldehydes. The most abundant aliphatic mono-unsaturated alkenes were C25∶1 and C14∶1. Fatty alcohols were mainly straight or mono-unsaturated, from 10 to 30 carbon-chain length. The most abundant alcohols were C28∶0 and C14∶0 chains, although unique mono-unsaturated alcohols with C16∶1 and C20∶1 were found in a few species. Among all of the saturated fatty alcohols, 1-nonasdecanol (C19), 1-pentacosanol (C25), and 1-triacontanol (C30) were unique to 1, 2, and 3 species, respectively. As shown in Tables 8 & 9, the fatty aldehydes, nonanal and octadecanal, were present in only 6 and 2 species, respectively.

### Sterols and Terpenes

The sterol composition of the pollen lipids was characterized by GC-MS, and it mainly consisted of β-sitosterol, [(3β-,24R)-ergost-5-en-3-yl]oxy], and stigmastan-3,5-diene, as judged by comparison of their mass spectra with known standards. These sterols were identified in 12-16 pollen species (see Table 10). In addition, stigmasterol and the tripene β-amyrin were present in 6 and 5 species of the pollen analyzed, respectively.

### Other Lipid Molecular Species

Numerous other lipid compounds have been identified in pollen lipophilic extracts; they differ considerably from one species to another, specifically, benzoic acid, glucitol, benzene, myo-inisitol, arabinitol, phenol, benzaldehyde, ribitol, inisitol, and cinnamic acid (see Tables 11 & 12). Phosphoric acid, phosphate, and pyrglutamate were the most abundant polar compounds found.

In general, most pollen species express discrete subsets of lipid components. A summary of the similarities, differences, and uniqueness of the pollen lipidome are shown in Tables 1,2,3,4,5,6,7,8,9,10,11,12, with the complete database for the 22 species available as supplemental data. Overall, more than 106 distinct lipid molecular species were identified unequivocally by retention times and mass spectral comparisons with the NIST/EPA/NIH library by use of AMDIS software. The GC-MS analysis revealed that the pollen grains harbor a plethora of lipid classes. In addition, there are many unique and common lipid molecular species in pollen grains.

As a starting point for the immunologic assessment of the lipid-regulating effects, we used lipids thought to be implicated in allergy and asthma through searches in PubMed. To investigate the pollen-lipid product activation of CD1d-resticted NKT cells, we used CD1d-resticted DN32.D3 hybridoma expressing an invariant NKT cell receptor incorporating the Vα14-Jα18 chain [31]. To that end, we developed an *in vitro* co-culture system using CD1d +/+ DC treated with lipids to stimulate mouse CD1d-resticted NKT cells. To explore the MyD88 dependence of the pollen lipid-induced immune response, we used WT.B6 and MyD88-deficient DCs after exposure of the cells to FAs, n-alkanes, sterols, or other lipids to examine their ability directly to stimulate the CD1d-reactive NKT cell line. When lipids-pulsed DCs were incubated with CD1d-resticted NKT cell, they showed stimulatory activity as determined by cytokine secretion.

**Figure 2:**
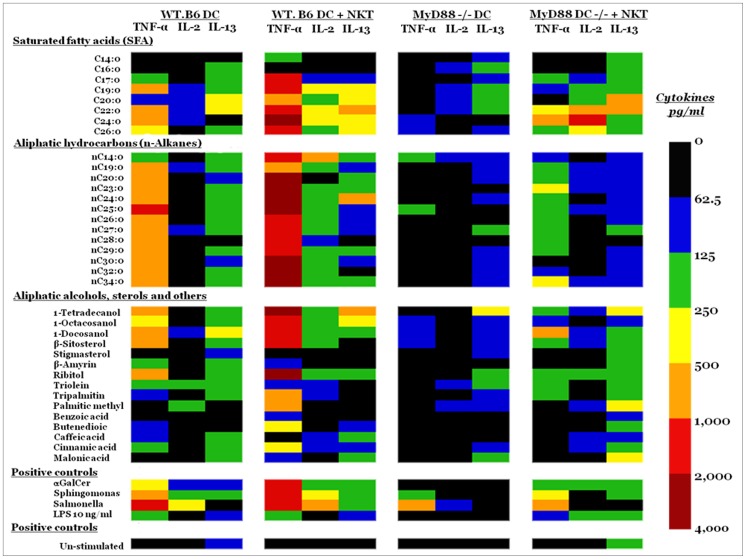
Heat map visualization comparing cytokine expression profiles of DC/NKT cells stimulated with lipid compounds *in vitro*. Several lipid compounds stimulated DC and DC/NKT cells and produced distinct cytokine patterns, including pro-inflammatory cytokines through toll-like receptor (TLR)-mediated DC activation. The co-culture with NKT cells augmented the inflammatory immune response to several lipid compounds. 1×10^5^ autologous immature dendritic cells (WT.B6 DC and MyD88^−/−^ DC) were left untreated or were stimulated with FAs and n-alkanes (1 µg/ml) or aliphatic alcohol, sterols, or other lipid compounds (5 µg/ml) or αGalCer (100 ng/ml) for 12 h in 96-well U-bottomed plates. Where indicated, 1×10^5^ purified NKT cells were added for an additional 36 h. Monocultures with DCs remained in culture for 48 hours. To exclude the possibility that the secreted cytokines are induced by entotoxin contamination, we measured endotoxin levels in pollen lipids by using limulus amebocyte lysate to confirm the absence of detectable levels of endotoxin. Pro-inflammatory (TNF-α) and pro-allergic (IL-13), regulatory (IL-10) and proliferatory (IL-2) levels in cell-free culture supernatants were then measured by use of ELISA. IL-10 is not shown in the heat map. The heat map represents ∼36 lipid compounds that are clustered into 4 groups based on their lipid classes (FAs, n-alkanes, alkanols, sterols, and controls) shown on the left of the heat map. Cytokines were clustered with the names shown on the top of the heat map. Each raw corresponds to a single lipid compound, and each column represents an independent condition. The heat map color scale corresponding to the relative expression of the cytokine relative to the minimum and maximum of all values is shown on the right. Black and blue indicating the lowest levels, brown and red indicating the highest levels, and green, yellow and orange indicating median levels relative expression of cytokines (average concentration pg/ml). Results are representative of two independent experiments.

Figure 2 presents a comparison of cytokine profiles of the most potent iNKT cell-stimulating pollen lipids. The heat-map graph of the cytokines indicates the distribution of signals in the two groups of WT.B6 and MyD88-deficient DCs mono-cultured or co-cultured with NKT. This visualization allows for comparisons of multiple signals in each treatment group. The pattern of cytokine increase was not uniform among individual DCs and/or NKT cells. Response of WT.B6 DC/NKT have different dynamic of cytokine production compared to MyD88-deficient DC/NKT. The heat map reveals that several FAs and n-alkanes induced strong pro-inflammatory cytokine responses by DCs/NKT. The production of the pro-inflammatory TNF-α by DC/NKT stimulated with n-alkanes is generally higher than that for FAs, but the pattern differs significantly among individual lipid molecules. When DCs pulsed with lipid compounds were co-cultured with mouse NKT cells, some lipids exhibited marked up-regulation of TNF-α as potent as α-GalCer in stimulating NKT cells.

The heat map also shows that some lipids produce lower levels of the T_H_2-associated (IL-13) from MyD88-deficient DCs in monoculture or co-cultured with NKT cells; however, notably higher IL-13 levels were induced when DC/NKT cells were stimulated with the saturated FAs C20∶0 and C22∶0. The heat map shows areas of relatively high (hot spots) and low (cold spots) cytokine expression in certain clusters of lipid compounds. The cytokine profiles clustering shows a clear, but incomplete separation of the stimulatory responses to various lipids. Results showed that the immune response was mostly derived from WT.B6 DCs co-cultured with NKT cells, and only medium to low levels of cytokines were secreted from DCs only. Our results showed that the MyD88 signaling pathway is required for the induction of a pro-inflammatory response to some lipid molecular species, and that NKT cells can augment the production of these inflammatory cytokines.

Stimulation of WT.B6 DC/NKT cells with C24∶0 and C22∶0 induced the greatest release of TNF-α (3750 and 1750 pg/ml, respectively), followed by C26∶0 and C17∶0. The results also show that FA C20∶0 activated MyD88−/− DC/NKT in co-culture and induced the T_H_2-associated IL-13 cytokine. Challenge with the FAs C24∶0 and C22∶0 increased the concentration of IL-13 in MyD88-deficient DCs, but to a significantly lesser extent than in wild-type DCs. Importantly, control FAs such as C10∶0 failed to do so (not shown). Addition of NKT cells to the DC culture further enhanced the production of T_H_2 cytokines, suggesting that DCs and NKT cells were engaged in an activation loop. Interestingly, MyD88 signaling seemed to be indispensable for IL-13 secretion by 20∶0, but not C22∶0, C24∶0, and C26∶0, suggesting that different FAs can use different signaling pathways. In contrast, co-culture of MyD88−/− DC with NKT showed marked production of IL-2 from C24∶0 and C22∶0. Stimulation of DCs/NKT with C24∶0 and C22∶0 also induced increased levels of IL-2 in WT.B6.

High levels of TNF-α (1000–3750 pg/ml) were observed in WT.B6 DC/NKT cells exposed to nC32, nC30, nC29, nC28, nC27, nC26, nC25, nC24, nC23, and nC14. In contrast, only exposure to nC24 induced a high concentration of IL-13 in cells from WT.B6 and not in MyD88-deficient cells. Moreover, the co-culture of WT.B6 DC and NKT in the presence of nC14 exhibited marked up-regulation of IL-2 relative to the control culture. An exaggerated release of TNF-α, IL-13, IL-10, and IL-2 following treatment with several n-alkanes was detected in the supernatant compared with controls. The trends for IL-10 secretion (not shown) by the NKT cells were similar to those observed for IL-2 secretion. These observations indicate that n-alkanes play a critical role in the induction of TNF-α and IL-13 from WT.B6 DC and NKT cells in the presence of MyD88 signaling.

Co-culture of WT.B6 DC and NKT cells with β-Sitosterol, Ribitol, and 1-Docosanol significantly induced high TNF-α levels, and the induction was higher than that of the positive control α-GalCer. Our results indicated that lack of the MyD88 signaling pathway markedly reduces TNF-α secretion.

**Figure 3:**
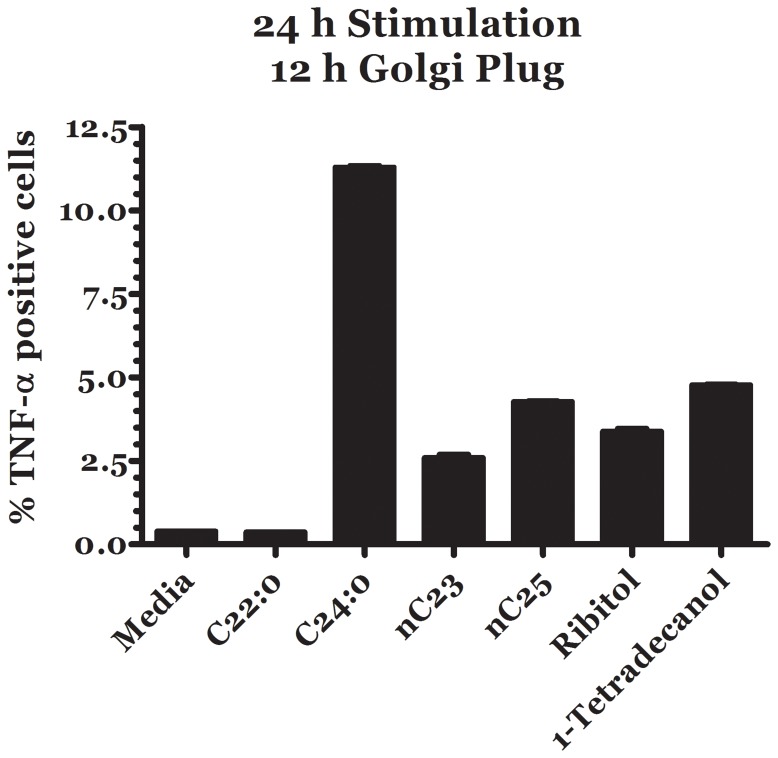
Effect of different lipid stimuli on TNF-α response of T cells. For analysis of cytokines by intracellular staining, conventional T cells harvested after culture *in vitro* were stimulated with 1 µg/ml lipid molecules for 24 to 36 hours in 6-well plates. After treatment with 10 µg/mL GolgiPlug, a protein transport inhibitor containing brefeldin A (eBiosciences) during the final 6 to 12 hours of stimulation, the cells were stained for surface markers and anti-mouse TNF-α as described in Materials and Methods. Flow cytometry was performed on a FACSCanto II instrument (BD Biosciences) and analyzed using FlowJo software (Tree Star).

We have used intracellular staining with cytokines combined with flow cytometry to examine the frequencies of TNF-α-producing cells from conventional T cells. FACS analysis showed that in the absence of lipids, 0.5% of unstimulated cells expressed intracellular TNF-α. This increased to 12% with 1 uM C24∶0, 2.5% with 1 uM nC23, 5% with 1 uM nC25 or 1-tetradecanol and 3.5% with 1 uM ribitol. Flow cytometric analysis showed several lipids found in pollen stimulate conventional T cells to express TNF-α (Figure 3).

## Discussion

The principal focus of this work is the characterization and distribution of the pollen lipid composition in 22 allergenic plant species and elucidation of the mechanism underling the rapid induction and expression of the immune response to pollen lipids. GC-MS analysis reveals differences in the content and distribution of lipid components among various pollen species. The pollen contains a complex mixture of aliphatic and aromatic compounds that belong to the classes FAs (saturated, unsaturated, and dicarboxylic), aliphatic hydrocarbons (n-alkanes and n-alkenes), fatty alcohols, sterols, and terpenes. Some of these compounds have been identified previously in particulate matter (aerosols) [34], [35], [36]. Because of their critical role in pollen-stigma interactions during sexual reproduction, lipids are found in the outermost layer of the pollen extracellular matrix (coat) [37].

In this study, we found that saturated and unsaturated FAs are among the compounds with the highest abundance and concentration in allergenic pollen. Saturated FAs and their metabolites regulate gene expression and immunologic pathways [38]. Palmitic (C16∶0), stearic (C18∶0), oleic C18∶1), and linoleic (C18∶2) acids regulate DNA synthesis and cytokine release in human peripheral lymphocytes *in vitro* [22], [23], [24], [25]. Palmitic acid induces production of the pro-inflammatory cytokine interleukin-8 [39] and IP-10, IL-8, MCP-1, COX-2, and MIG expression in human macrophages via NF-κB activation [40]. Here, we showed that C20∶0 and C22∶0-induced DC/NKT stimulation, and caused rapid, substantial and specific up-regulation of T_H_2-associated IL-13 from MyD88 deficient DCs/NKT. Although the nature of the T_H_2-inducing pattern recognition receptors is unknown, DC/NKT activation in the absence of MyD88 suggest the presence of toll-like receptor (TLR)-independent pattern-recognition receptor in T_H_2-induction against C20∶0 and C22∶0.

The secretion of IL-13 is functionally important, because IL-13 binds to the α-chain of the IL-4 receptor and is expressed by T_H_2 cells in allergic patients. IL-13 is associated with the induction of polarization toward a T_H_2-IgE-mediated allergic response. The secretion of T_H_2-biased immune response from pollen lipids via NKT cells is consistent with the established function of NKT cells in driving the acquired immune response. Taking this into account, it seemed likely that IL-13 secretion may be particularly important during early events of T_H_2 polarization and initiation of an allergic response after pollen inhalation. The mechanism that can account for the cytokine milieu during allergenic protein presentation during the sensitization phase is very critical. Release of IL-13 during the initiation of the allergic response could rapidly influence DC differentiation to secrete T_H_2-promoting factors (DC2s), which then promote activation of CD1d-resticted iNKT cells to produce more T_H_2 cytokines. These iNKT cells, in turn, will further activate DC2s and promote the differentiation of T_H_2 cells, leading to IgE secretion in the presence of allergenic pollen proteins.

Our results are in agreement with previous reports showing that monocyte-derived dendritic cells that matured in the presence of long-chain FAs lack IL-12 production, and direct the differentiation of T_H_ cells toward the T_H_0/T_H_2 phenotype [41]. Naturally occurring PPARγ ligands, like FAs and FAs derivates, also have T_H_2 anti-inflammatory effects by redirecting DCs into a less stimulatory mode [42]. PPARγ is important in the control of asthma, allergy, and airway inflammatory responses and the modulation of allergic inflammation through up-regulation of PTEN (phosphatase and tensin homolog) [43], [44], [45], [46], [47]. The inflammatory and modulatory potential of long-chain FAs present in the pollen of several allergenic species may be a crucial factor in pollen-induced airway inflammation and allergic immune sensitization to pollen.

Our data show that n-alkanes activate NKT cells and promote DC/NKT cells to secrete the pro-inflammatory cytokine TNF-α. The production of large quantities of TNF-α by Vα14 iNKT cells can also exacerbate the development of airway inflammation in asthma. Several pollens showed the presence of n-alkanes nC14–nC22 as trace constituents, as well as the more abundant nC27, nC25, and nC29. Lipids identified in pollen stimulated the production of TNF-α and the highest frequencies of TNF-α were seen for FA C24 followed by 1-tetradecanol and nC25. These data make an excellent case for the idea that NKT cells help drive the acquired immune response. The secretion of pro-inflammatory cytokines by cells stimulated with n-alkanes is further supported by evidence implicating aliphatic hydrocarbons in enhancing immune responses [26], [48], [49]. Hydrocarbons nC12 and nC14 raise the concentrations of the important inflammatory-response markers in plasma, namely, sialic acid and creatine kinase, immediately after administration [50]. The n-alkanes nC12 and nC14 are among the hydrocarbons identified in pollen investigated in this study that may accentuate the immune-inflammatory response.

Among the sterols present in pollen lipids are β-sitosterol and stigmasterol, which have been identified as the causal agents for asthma epidemic outbreaks [51], [52]. Several volatile organic compounds (VOCs) were detected in pollen, including benzene, benzaldehyde, n-decane, n-dodecane, nonanal, phenol, fatty aldehydes, and fatty alcohols. It was reported that VOCs cause airway diseases that induce pathology similar to that of asthma and bronchitis; they have also been associated with an increased incidence of obstructive airway disease [53]. This is consistent with the finding that VOC emitted from *Brassica napu* (rapeseed) fields during the flowering period may be causative factors of rapeseed allergy/toxicity [54]. NKT cells are critical components of the adjuvant network that primes adaptive immune responses. Pollen Lipids can also affect allergic response, for instance by activating NKT cells and enhancing the response of other immune cells, thus acting as an adjuvant for the pollen allergenic proteins. In summary, pollen-derived lipids regulate immune response to pollen in a pathway that involves NKT cells.

Pollen allergy is acknowledged as a major risk factor for asthma. There is now clear evidence that asthma is a chronic inflammatory disorder of the airways driven by a T_H_2 allergic response to innocuous airborne environmental antigens in cooperation with inflammatory cells as distinctive constituents of the inflammatory infiltrate [55]. The allergic components of asthma are treated with anti-IgE drugs [56], whereas the inflammatory components are treated with anti-inflammatory drugs, i.e., corticosteroids, anti-leukotrienes, and leukotriene-modifying agents [57]. Here, we show the presence of several pollen-derived lipids with pro-allergic, pro-inflammatory and immunomodulatory properties highlighting their immunologic activities on the allergen presenting cells (APCs) and NKT cell responses. By virtue of their location on the surface of pollen, lipid components may play an active role in many facets of the immune response to pollen, such as the active recruitment of a wide array of inflammatory mediators.

We propose that lipids direct the allergic response by controlling the early immune response signals, and that they amplify the response of allergic proteins, yielding a potent effector allergic function. This indicates that lipids are an essential factor needed for polarization toward a T_H_1, T_H_2, or regulatory response, and that, in the presence of these lipids, pollen allergenic proteins may be able to generate an allergic or inflammatory response. This supports the view that the pollen lipids play a vital role in the host defense against pollen by elaboration of bioactive lipid mediators [16], [17], [18], [19], [20], influencing the inflammatory aspects of allergic asthma. The control that lipids exert on the immune response depends on the characteristics of the lipid.

The series of potential biomarkers identified in the present study provided some insight into the number, identity, and relative levels of pollen lipids in allergenic pollen plant species. Pollen lipids contain a number of compounds that have been identified previously to act as immune stimulants or enhancers of allergic and inflammatory responses. Based on the present results from GC-MS analysis and previous studies, we suggest that the rich content of FAs, n-alkanes, and sterols in the pollen in combination with antigenic proteins is probably what makes pollen a strong immune stimulant. Taking this into account, it seems likely that pollen lipids have signaling function. It remains to be explored whether the synergistic combinations of allergenic protein-loaded pollen and aliphatic hydrocarbons and/or FAs found on the pollen may increase the allergenic potency of pollen grains.

In conclusion, the current GC-MS lipid-profiling data on allergenic pollen and the *in vitro* studies on selected pollen-derived lipids suggest a causal relationship between lipid compounds and inflammatory and modulatory responses. Furthermore, the lipid-profile data seem to have important implications for study of the mechanisms underlying the early events of allergic response and the design of assays for predicting the sensitization potential of pollen lipids. The picture emerging from this study proposes that pollen is a source of both bioactive lipids and allergenic proteins, and that it might thus have a broader role in stimulating immune hyperreactivity than was previously appreciated. Further investigations are ongoing for verifying and confirming the allergenic and inflammatory nature of the lipophilic constituents of pollen.

The data presented here show that pollen-lipid molecular species have the potential to stimulate immune responses. It thus seems that pollen lipids alter the expression of signaling molecules secreted by DC/NKT cells that actively stimulate the development of allergic and inflammatory immune responses. Together, these results suggest that pollen lipids possess a multidimensional capacity to stimulate the immune cells, perhaps through their innate ability to promote a cellular response and enhance the allergic phenotype.

Further research should be done for finding out the role and mechanism of pollen lipid molecular species and their interactions with pollen proteins or other small molecules. This will be very helpful for research on allergic pathogenesis and for finding a new drug target. The identified potential biomarkers may be the target components in future mechanistic studies. This work forms a necessary part of an ongoing series of investigations for establishing whether any immunologic role of pollen can be attributed to lipids, and the type, nature, and extent of that role. Characterizing the contingent of pollen lipids and their abundance and how this is associated with the allergic immune response will facilitate our understanding of how to intervene to correct allergic responses augmented by pollen lipids.

## Supporting Information

Table S1 (XLS)

Table S2 (XLS)

Table S3 (XLS)

Table S4 (XLS)
